# Supplementary material for: Soil Rehabilitation Promotes Resilient Microbiome with Enriched Keystone Taxa than Agricultural Infestation in Barren Soils on the Loess Plateau
Source: Biology (Basel). 2021 Dec 2;10(12):1261. doi: 10.3390/biology10121261 (PMC8698737; doi:10.3390/biology10121261)
Supplement: Supplementary file 1 [file biology-10-01261-s001.zip › biology-1455643-supplementary-figure.pdf]

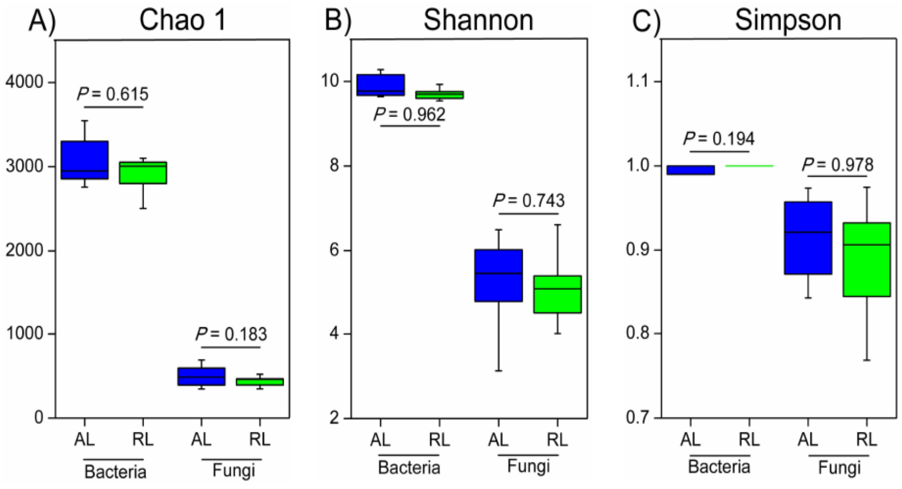

Figure S1. Bacterial and fungal diversity indices in agricultural (AL) and rehabilitated lands (RL). Chao 1 index indicate microbial richness, Shannon index indicate microbial diversity and Simpson index indicate microbial evenness as a measure of microbial alpha diversity. For individual index boxes, significant differences between the land-use types (AL and RL) were tested by independent sample Wilcoxon test (n = 9 in each group).
